# Atypical cortical thickness and folding of language regions in Chinese nonsyndromic cleft lip and palate children after speech rehabilitation

**DOI:** 10.3389/fneur.2022.996459

**Published:** 2022-09-20

**Authors:** Shi Wang, Lei Fang, Guofu Miao, Zhichao Li, Bo Rao, Hua Cheng

**Affiliations:** ^1^Department of Neonatology, Wuhan Children's Hospital (Wuhan Maternal and Child Healthcare Hospital), Tongji Medical College, Huazhong University of Science and Technology, Wuhan, China; ^2^Department of Nuclear Medicine, Wuhan Children's Hospital (Wuhan Maternal and Child Healthcare Hospital), Tongji Medical College, Huazhong University of Science and Technology, Wuhan, China; ^3^Department of Rehabilitation Medicine, Zhongnan Hospital of Wuhan University, Wuhan University, Wuhan, China; ^4^Department of Rheumatism Immunology, Wuhan Children's Hospital (Wuhan Maternal and Child Healthcare Hospital), Tongji Medical College, Huazhong University of Science and Technology, Wuhan, China; ^5^Department of Radiology, Zhongnan Hospital of Wuhan University, Wuhan University, Wuhan, China; ^6^Department of Radiology, Beijing Children's Hospital, Capital Medical University, National Center for Children's Health, Beijing, China

**Keywords:** nonsyndromic cleft lip and palate, speech therapy, cortical thickness, gyrification index, surface-based morphometry

## Abstract

**Objective:**

Even after palatoplasty and speech rehabilitation, patients with cleft lip and palate (CLP) remain to produce pronunciation errors. We hypothesized that nonsyndromic CLP (NSCLP) after speech rehabilitation had structural abnormalities in language-related brain regions. This study investigates structural patterns in NSCLP children after speech rehabilitation using surface-based morphometry (SBM) analysis.

**Methods:**

Forty-two children with NSCLP and 42 age- and gender-matched healthy controls were scanned for 3D T1-weighted images on a 3T MRI scanner. After reconstructing each brain surface, we computed SBM parameters and assessed between-group differences using two-sample *t*-tests and permutation tests (5,000 times). Then, we assessed the relationship between the SBM parameters and the Chinese language clear degree scale (CLCDS) using Pearson's correlation analysis.

**Result:**

The speech-rehabilitated children with NSCLP showed lower cortical thickness and higher gyrification index mainly involving left language-related brain regions (permutation tests, *p* < 0.05). Furthermore, the lower cortical thickness of the left parahippocampal gyrus was positively correlated with CLCDS scores (*r* = 0.370, *p* = 0.017) in patients with NSCLP.

**Conclusion:**

The SBM analysis showed that the structural abnormalities of speech-rehabilitated children with NSCLP mainly involved language-related brain regions, especially the dominant cerebral hemisphere. The structural abnormalities of the cortical thickness and folding in the language-related brain regions might be the neural mechanisms of speech errors in NSCLP children after speech rehabilitation. The cortical thickness of the parahippocampal gyrus may be a biomarker to evaluate pronunciation function.

## Introduction

Cleft lip and palate (CLP) is one of the most common craniofacial malformations in infants and is divided into syndromic CLP and nonsyndromic CLP (NSCLP) according to whether the CLP is part of a well-known syndrome (1). NSCLP accounts for 70% of CLP with unclear etiology. The incidence rate of speech disorder, the most common complication of NSCLP, ranges from 22 to 92% (2). Even with early surgical treatment, 30–50% of CLP patients still suffered “cleft palate speech” characterized by hypernasality and/or nasal emission (3). Therefore, cleft repair and speech therapy are the most common methods of cleft palate management (4). However, studies confirmed the residual speech disorders in CLP patients with postsurgical repair and speech therapy, and the percentage of consonant errors ranged from 15 to 22% (5–8). Studies have confirmed brain structural differences in brain regions for patients with CLP before surgery and speech training (9, 10). Thus, we hypothesized that patients with NSCLP after surgery and speech therapy had structural abnormalities in language-related brain regions.

Voxel-based morphometry (VBM) analysis, a common method of assessing brain structure, has confirmed that the gray matter volume was lower in the frontal lobe and higher in the temporal lobe in patients with CLP (11). Besides, gray matter density was higher in the left superior temporal gyrus and fusion (12) and was lower in the bilateral medial frontal cortex (10). Different from VBM analysis, surface-based morphometry (SBM) analysis could measure the cortical thickness (CT) and gyrification index (GI) of every cortical region. A neuroimaging study found that cortical thickness was higher in left pars opercularis and triangularis in CLP children than in healthy peers (13), and cortical folding also changed in adult patients with CLP before and after rehabilitation. Li et al. (14) found that the changes in cortical thickness and gyrification occurred in brain regions related to language, execution, and auditory functions in NSCLP children. However, the abnormalities in the SBM patterns are unclear in NSCLP children after speech rehabilitation.

We hypothesized that regional brain structural abnormalities in the NSCLP children after speech therapy might lead to residual speech errors. Therefore, this study investigated the possible morphological pattern of the cerebral cortex in speech-rehabilitated children with NSCLP using the SBM analysis.

## Materials and methods

### Participants

The ethical committee of the Beijing Children's Hospital approved this study, and informed consent was obtained from all subjects and their legal guardian. A total of 42 children (31 boys and 11 girls) with NSCLP (NSCLP group) and 42 age- and gender-matched typical developing healthy controls were recruited for the current study from the Beijing Children's Hospital. The inclusion criteria were as follows: (1) NSCLP patients (ranging from 6 to 16 years) who started the speech therapy 3–6 months after a successful pharyngeal closure surgery, by the frequency of 30 min/day and 3 times/week, lasting for half a year, till they reached the basic point 86 according to the Chinese language clear degree scale (CLCDS) scores; (2) normal vision and hearing (by auditory brainstem response examination below 30 dB nHL); (3) average intelligence [more than 90 scores using the Chinese Wechsler Intelligence Scale for Children-IV (CWISC-IV)]; and (4) right-handed subjects. The exclusion criteria of the patients were as follows: NSCLP patients with clinic diagnoses of (1) dysarthria; (2) velopharyngeal insufficiency; (3) hearing and/or vision impairments; (4) developmental delays; (5) congenital disorders; (6) other syndromes or possible; and (7) other chronic health diseases.

### Image acquisition

All data were acquired on a 3.0 T GE MRI system at the Department of Radiology (Beijing Children's Hospital). For each subject, high resolution 3D T1-weighted gradient-echo images were acquired with the following parameters: repetition time (TR) = 8.2 ms, echo time (TE) = 3.5 ms, inversion time (TI) = 450 ms, flip angle (FA) = 13°, matrix = 256 × 256, and FOV = 256 × 256, 164 continuous sagittal slices were scanned to cover the whole brain, and slice thickness = 1 mm.

### Surface-based morphometry analysis

Cortical thickness and gyrification index are widely used in the SBM analysis of structural plasticity. Cortical thickness is a sensitive metric of dynamic brain alterations across development stemming from maturational pruning and experiential neuroplasticity (15), and the gyrification index provides a new method for analyzing brain structure. It focuses on the frequency of gyrus folding, describing either structurally constrained cognitive characteristics or acquired long-term experience-dependent plasticity (16). Moreover, because the gyrification index is characterized by relative lifetime stability, it is suitable as an index of aberrant neurodevelopment (17).

For the SBM analysis, the CAT12 (Computational Anatomy Toolbox, http://dbm.neuro.uni-jena.de/cat/) and SPM12 (statistical parametric mapping, http://www.fil.ion.ucl.ac.uk/spm/) were used within the MATLAB environment. We used this software to reconstruct the cortical surface of each subject and calculate multiple morphometric parameters (18, 19). During the procedure, cortical thickness estimation and the central surface were carried out in one step based on the projection-based thickness (PBT) approach (19). Then, the topology correction, spherical mapping, and spherical registration were performed for cortical thickness and central surface. After that, the gyrification index was extracted from the central surface. Finally, the surface of cortical thickness and gyrification index was resampled and smoothed with a 15-mm filter for each hemisphere. For further statistical analysis, the mean cortical thickness and gyrification index were extracted for 68 ROIs defined by the Desikan-Killiany atlas (20) with CAT12.

### Statistical analysis

Statistical analyses were conducted using the statistical module CAT12/SPM12. The age and gender were taken as covariates, and the between-group differences in the mean regional value of the two morphometric parameters were tested with two-sample *t*-tests (*p* < 0.05) and permutation tests 5,000 times (*p* < 0.05). In addition, the mean values of between-group different regions were calculated for the correlations between the metrics and CLCDS in the NSCLP group using Pearson's correlation analyses (*p* < 0.05).

## Results

### Demographic data

Table 1 shows that it was not significant in the age (*t* = −0.46, *p* = 0.96), CWISC-IV scores (*t* = −1.18, *p* = 0.24), and education years (*t* = −1.12, *p* = 0.28) of children between the NSCLP group and healthy controls. The CLCDS scores were 91.45 ± 3.92 in the NSCLP children. The number of boys and girls showed no between-group significant differences in the two groups (χ^2^ = 0, *p* = 1).

**Table 1:** Demographic data.

	**Age (years)**		**Boys/girls**	**CLCDS scores**	**CWISC-IV scores**	**Education (years)**
**Sample members**	**Mean ±SD**	**Median**	**No**.	**Mean ±SD**	**Mean ±SD**	**Mean ±SD**
NSCLP children	9.71 ± 2.31	10	31/11	91.45 ± 3.92	97.5 ± 9.5	4.0 ± 2.2
Healthy controls	9.68 ± 2.21	10	31/11	–	99.8 ± 6.6	4.7 ± 2.3

CLCDS, Chinese language clear degree scale; NSCLP, nonsyndromic cleft lip and palate; CWISC-IV, Chinese Wechsler Intelligence Scale for Children-IV; SD, standard deviation; No., number.

### The lower cortical thickness in the NSCLP group

**Figure 1:**
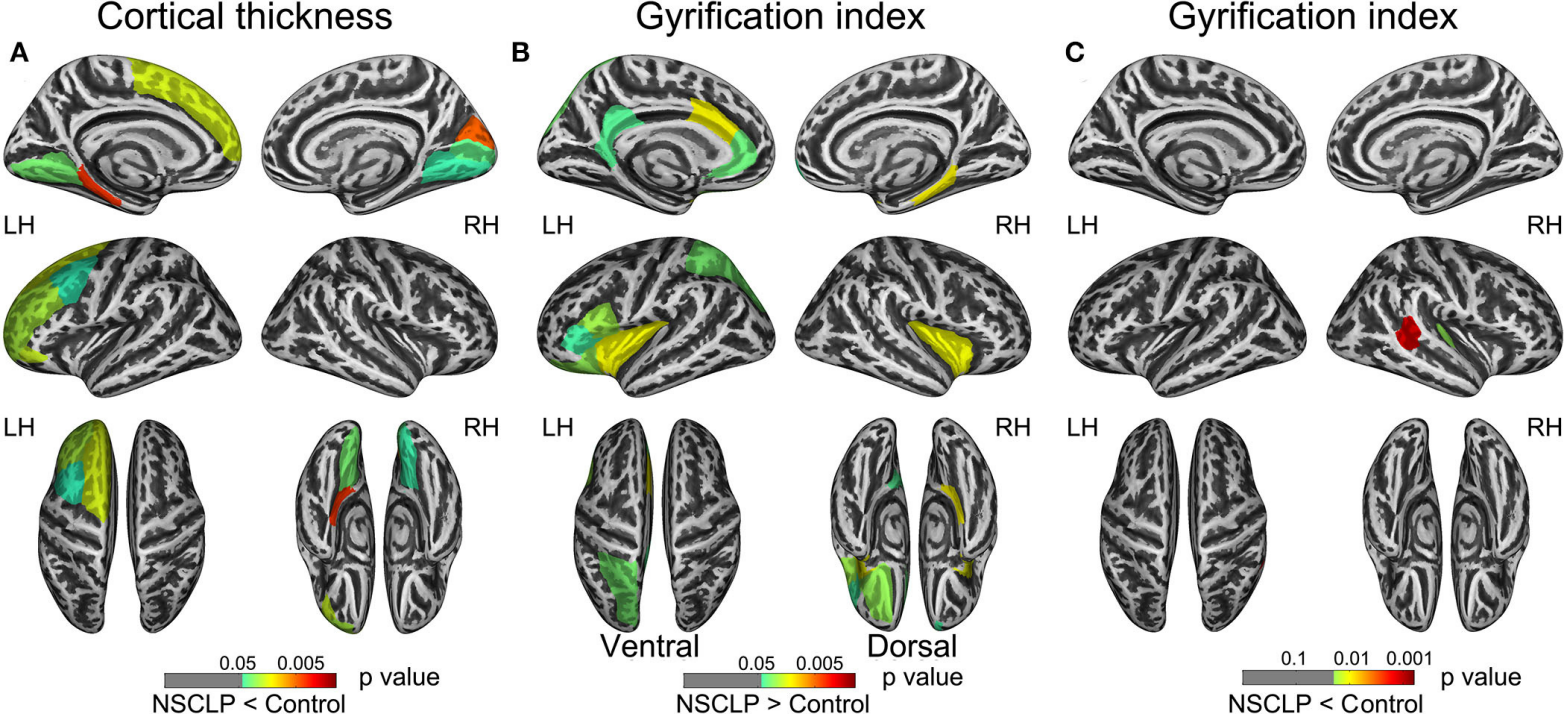
shows regions with a significant between-group cortical thickness (CT) and gyrification index (GI). **(A)** The distribution of regions with lower CT showed in the NSCLP patients. **(B,C)** The distribution of higher GI **(B)** and lower GI **(C)** regions showed in patients with NSCLP. The color bars indicated the *p-*value. Two-sample *t*-test (*p* < 0.05) and permutation tests 5,000 times (*p* < 0.05). Corrected for age and gender. NSCLP, nonsyndromic cleft lip and palate; LH, left hemisphere; RH, right hemisphere.

**Figure 2:**
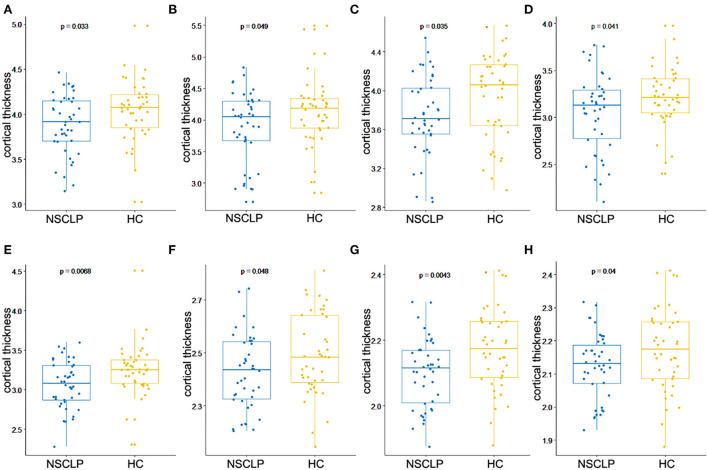
Bar plot for cortical regions showed significant between-group differences in the cortical thickness, including left superior frontal gyrus **(A)**, left middle frontal gyrus **(B)**, left pars orbitalis of inferior frontal gyrus **(C)**, left lingual gyrus **(D)**, left parahippocampal gyrus **(E)**, right lingual gyrus **(F)**, right pericalcarine **(G)**, and right cuneus **(H)**. The blue color represents NSCLP, and the yellow color represents HC (healthy controls).

Figure 1A depicts a whole-brain cortical thickness analysis comparing healthy controls to the NSCLP group, controlling for age and gender with permutation tests (*p* < 0.05). Compared with healthy controls, the cortical thickness in the bilateral lingual gyrus was lower in the NSCLP group. The thinner cortex was found in additional left hemisphere areas, including the superior/middle frontal and parahippocampal gyrus. In the right hemisphere, the regions of the thinner cortex in the NSCLP group included pericalcarine and cuneus cortices. The size (mm^2^) of each cluster and the location coordinated at Montreal Neurological Institute (MNI) are shown in Table 2. Compared to healthy controls, the bar plot graph of each cluster with thinner cortex in the NSCLP group was demonstrated in Figure 2.

**Table 2:** Descriptive report of brain regions showing a significant difference (*P* < 0.05) between patients with NSCLP and healthy controls on cortical thickness and gyrification index.

	**Voxel size (mm^2^)**	***t***	***p***	**Mean metric (SD)**	
				**NSCLP**	**Control**
**Cortical thickness (mm)**					
Left superior frontal	12,298	−2.172	0.033	3.88 (0.33)	4.05 (0.34)
Left caudal middle frontal	10,851	−2	0.049	3.92 (0.60)	4.13 (0.52)
Left pars orbitalis	965	−2.142	0.035	3.74 (0.41)	3.94 (0.45)
Left lingual	4,229	−2.077	0.041	3.06 (0.43)	3.22 (0.32)
Left parahippocampal	1,812	−2.778	0.007	3.07 (0.30)	3.26 (0.32)
Right lingual	3,894	−2.008	0.048	2.43 (0.15)	2.49 (0.15)
Right pericalcarine	1,837	−2.084	0.04	2.12 (0.13)	2.18 (0.13)
Right cuneus	1,616	−2.668	0.009	2.25 (0.12)	2.32 (0.12)
**Gyrification index (%)**					
Left insula	5,249	2.251	0.027	25.85 (1.21)	25.31 (0.93)
Left lateral orbitofrontal	4,229	2.218	0.034	26.34 (1.28)	25.82 (1.13)
Left pars triangularis	3,062	2.026	0.046	27.33 (1.07)	26.84 (1.49)
Left pars opercularis	2,041	2.09	0.04	26.35 (1.09)	25.88 (1.07)
Left superior parietal	10,456	2.076	0.043	29.65 (0.98)	29.24 (1.01)
Left caudal anterior cingulate	1,450	2.276	0.025	25.10 (2.22)	24.13 (1.68)
Left rostral anterior cingulate	1,338	2.065	0.048	25.71 (2.80)	24.73 (1.99)
Left isthmuscingulate	2,524	2.089	0.04	25.17 (2.91)	24.24 (1.69)
Right insula	5,046	2.237	0.028	26.69 (1.01)	26.14 (1.21)
Right parahippocampal	1,728	2.282	0.026	24.50 (3.12)	23.29 (1.43)
Right caudal superior temporal	2,196	−3.162	0.001	25.22 (1.73)	26.36 (1.60)
Right transverse temporal	781	−2.089	0.04	22.27 (2.68)	23.47 (2.57)

Two-tailed statistical t-values of the mean regional cortical thickness and gyrification index between the two groups (*p* < 0.05). Corrected for age and gender with permutation tests 5,000 times (*p* < 0.05). NSCLP, nonsyndromic cleft lip and palate; L, left; R, right.

### The higher gyrification index in the NSCLP group

**Figure 3:**
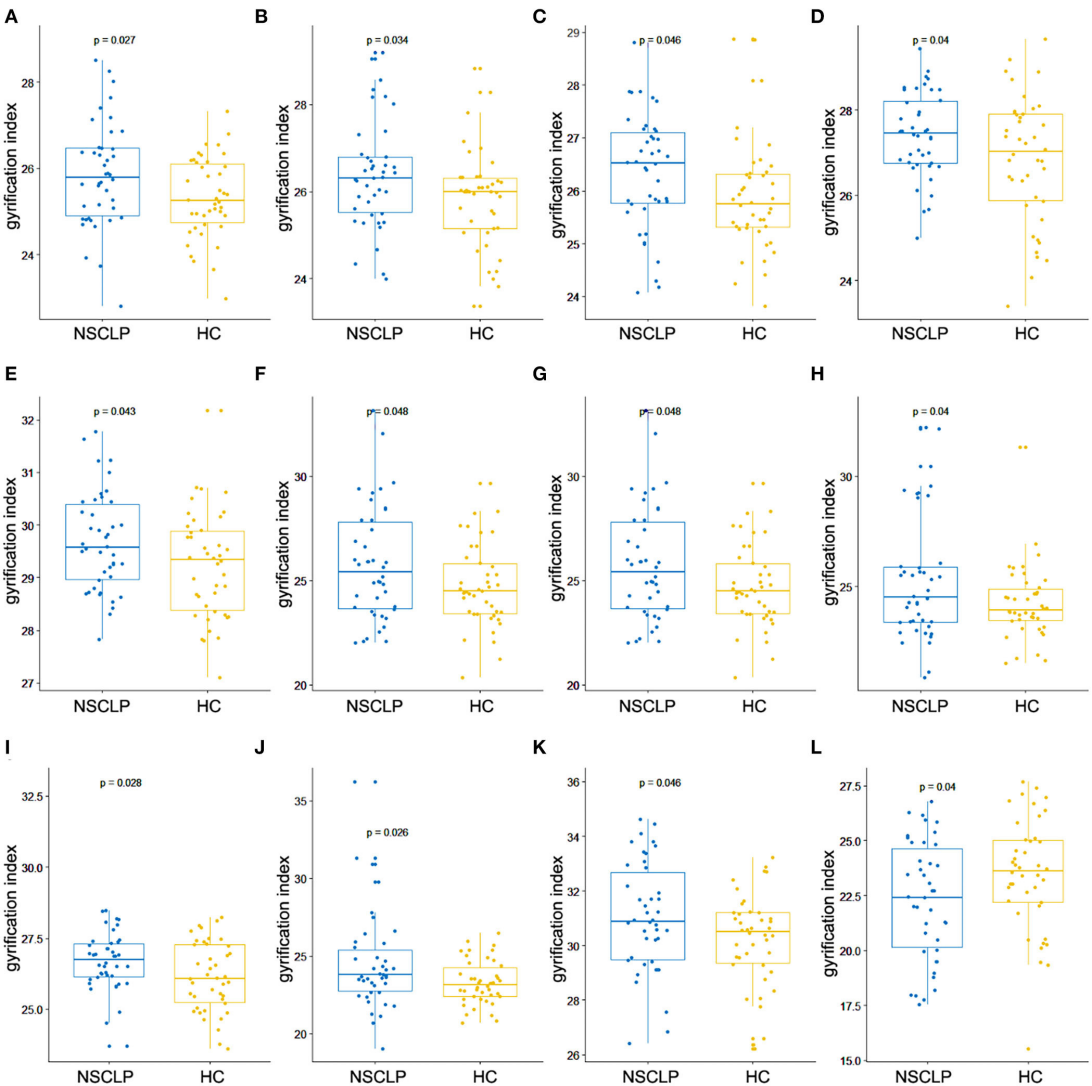
Bar plot for cortical regions showed significant between-group differences in the gyrification index. Left insula **(A)**, left lateral orbitofrontal **(B)**, left pars triangularis **(C)**, left pars opercularis **(D)**, left superior parietal lobule **(E)**, left rostral anterior cingulated **(F)**, left caudal anterior cingulated **(G)**, left isthmus cingulate **(H)**, right insula **(I)**, right parahippocampal **(J)**, right caudal superior temporal **(K)**, and right transverse temporal gyrus **(L)**. The blue color represents NSCLP, and the yellow color represents HC (healthy controls).

Figures 1B,C depicts whole-brain regional gyrification index analysis comparing healthy controls to the NSCLP group, controlling for age and gender with permutation tests (*p* < 0.05). The gyrification index of the bilateral insula gyrus, the right parahippocampal gyrus, and the left hemisphere (lateral orbitofrontal, pars opercularis, pars triangularis, superior parietal, and cingulate gyrus) were higher in the NSCLP group than in healthy controls. Compared to healthy controls, a lower gyrification index was shown in the NSCLP group on the right hemisphere transverse temporal and caudal superior temporal gyrus (*p* < 0.05). The size (mm^2^) of each cluster and the location coordinated at Montreal Neurological Institute (MNI) were shown in Table 1. Compared to healthy controls, the bar plot graph of each cluster with a different gyrification index in the NSCLP group was demonstrated in Figure 3.

### Correlations between regional structural differences and CLCDS

**Figure 4:**
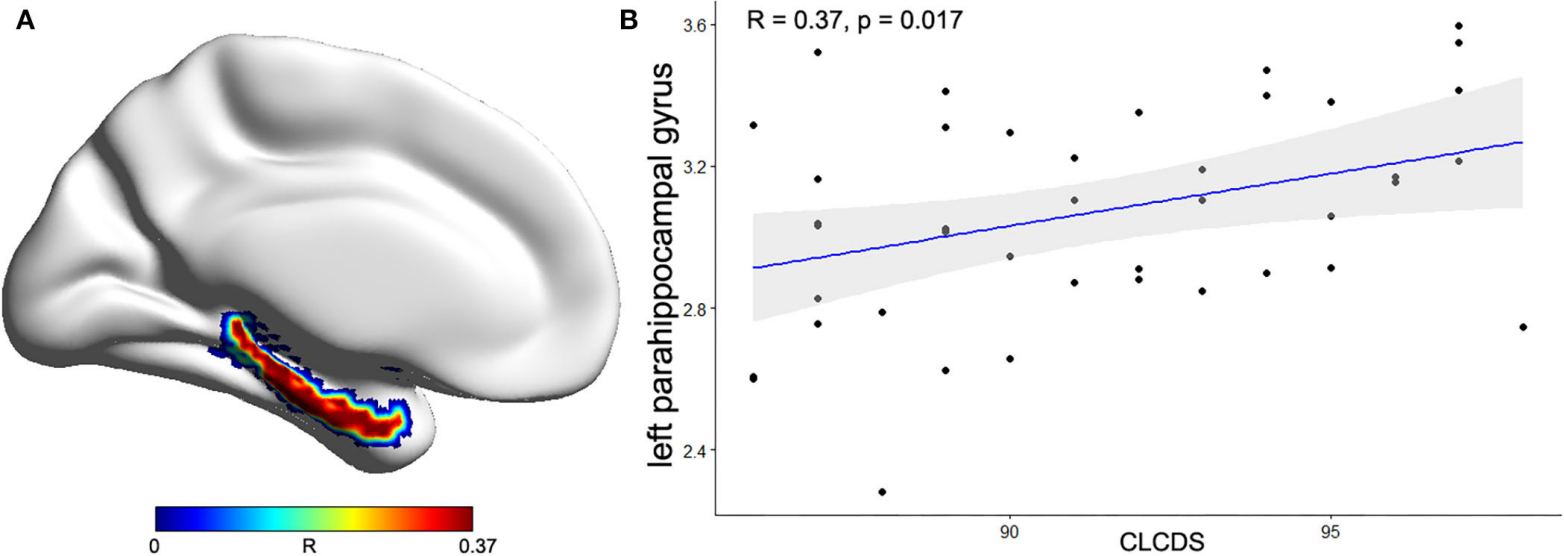
**(A)** The correlation coefficient of the left parahippocampal gyrus between the mean cortical thickness (CT) and CLCDS scores was projected onto the cortical surface. **(B)** The positive correlation between the mean CT of the left parahippocampal gyrus and CLCDS scores in the NSCLP group. The color bars indicated R-value. CLCDS, Chinese language clear degree scale.

The correlations between the cortical morphological parameters and CLCDS scores were calculated, finding a positive correlation between the cortical thickness of the left parahippocampal gyrus and CLCDS scores (*r* = 0.370, *p* = 0.017) in the NSCLP group (Figure 4).

## Discussion

This study investigated the surface-based morphological patterns of patients with NSCLP after speech rehabilitation. Compared with healthy controls, the differences in cortical thickness and gyrification index were identified primarily in the language-related brain regions in speech-rehabilitated patients with NSCLP. Furthermore, the cortical thickness of the left parahippocampal gyrus showed a positive correlation with the CLCDS scores.

### The differences in cortical thickness

Consistent with our hypothesis, we identified that the residual speech errors in NSCLP children after speech rehabilitation demonstrated a Thinner cortex than healthy controls in language-related brain areas, including the left superior frontal gyrus, left caudal middle frontal, left pars orbitalis, left parahippocampal, right pericalcarine, right cuneus, and bilateral lingual. The left superior frontal and middle frontal gyrus were crucial areas of the dorsal phonological processing stream (21). At the same time, the cuneus, pericalcarine cortices, and lingual gyrus were essential brain regions in the ventral visual pathway (22) and the reading circuitry (23). The parahippocampal gyrus is involved in increasing expertise (24, 25). Previous studies confirmed that expertise training, e.g., simultaneous interpretation learning (26) and diving training (25), could increase cortical thickness. Our result found that the cortical thickness of the language-related brain areas in NSCLP children after speech therapy still did not reach an average level, which indicated that the speech-rehabilitated children with NSCLP also had structural brain differences.

### The development patterns of the gyrification index in school-age children

The declining period of the gyrification index describes the fine-tuning process during the school-aged child period for cortical maturation (27). In this period, a previous study confirmed that developmental dyslexia could lead to an increased gyrification index (28). Moreover, expertise training induces the deceased gyrification index, such as diving training (29).

### The differences in the gyrification index

The higher gyrification indexes were detected in the insula, lateral orbitofrontal cortices, pars opercularis, pars triangularis, superior parietal lobule, and cingulate gyrus of the left hemisphere and in the insula and parahippocampal gyrus of the right hemisphere. The pars triangularis and pars opercularis belong to Broca's area, which was related to the motor function of language (30). In our study, all subjects were at age 6–16. Thus, they were in the declining period of the gyrification index. The higher gyrification index of these language-related brain regions suggested immature neural circuitry, which might result from the speech dysfunctions of NSCLP children. Our result showed that the higher gyrification index did not develop to normal levels in NSCLP children after speech therapy, indicating structural-developmental disorders.

The insular cortex, lateral orbitofrontal cortices, pars opercularis, pars triangularis, and superior parietal lobule are associated with phonological processing (21, 31). The cingulate gyrus is concerned with assessing the spatial, context, and personal relevance of sensory information (32). Therefore, this abnormal brain structural development may be closely related to speech disorders. A study found increased gyrification of parietal, frontal, and temporal lobes in adults with NSCLP after articulation rehabilitation (14), similar to our research.

Moreover, the lower gyrification indexes were located in the right transverse and caudal superior temporal gyrus. The caudal superior temporal belongs to Wernicke's area, the sensory language center (33). The transverse temporal gyrus is involved in acoustic signal perception (34). Besides, the caudal superior temporal gyrus is demonstrated to transfer language sound into phonemes from the transverse temporal gyrus (21). The decreased gyrification index suggested that neural connections in these regions were strengthened for cortical maturation (27). Therefore, we could infer that not only the earliest sound process of the right transverse temporal and causal superior temporal gyrus was not impaired, but also more sound information of speech therapy through these brain regions led to the neuroplasticity, describing the lower gyrification index.

### Behavioral correlation

Additionally, the correlation analysis showed that the mean cortical thickness of the left parahippocampal gyrus was positively correlated with the CLCDS scores. The parahippocampus, an important memory brain region, receives input from other brain regions and processes several types of sensory information (35). Li et al. (14) found that changed attention and language networks and working-memory brain structures (hippocampal and parahippocampal gyrus) were related to impaired language function. Our results suggested that the cortical thickness in the left parahippocampal gyrus was closely related to pronunciation function in the NSCLP children, indicating the state of pronunciation function. Furthermore, the mean cortical thickness of the left parahippocampal gyrus was lower in the NSCLP group than in healthy controls, which suggested that the damage to the parahippocampal gyrus might prevent the pronunciation function recovery. The lower cortical thickness of the left parahippocampal gyrus might be underlying neural mechanisms in NSCLP children after speech therapy, which might be a biomarker for evaluating pronunciation function.

## Limitations

This study still has some limitations. First, more subjects should be enrolled in further studies. Second, more work focused on the investigation of the differences in both functional and structural abnormalities for CLP children before speech therapy should be done in the future. Third, the CLP patients with special consonant pronunciation errors, such as affricates, would be further estimated for the changes in the brain structures in the future.

## Conclusion

In our study, the speech-rehabilitated children with NSCLP had speech errors clinically and neuroimaging abnormalities structurally. The structural impairments of cortical thickness and folding in the language-related brain regions might be the neural mechanisms of speech errors in NSCLP children after speech rehabilitation. Moreover, the cortical thickness of the left parahippocampal gyrus might indicate pronunciation function in NSCLP children after speech rehabilitation.

## Data availability statement

The original contributions presented in the study are included in the article/supplementary material, further inquiries can be directed to the corresponding author/s.

## Ethics statement

The studies involving human participants were reviewed and approved by the Ethical Committee of the Beijing Children's Hospital. Written informed consent to participate in this study was provided by the participants' legal guardian/next of kin.

## Author contributions

SW: conceptualization and writing—reviewing and editing. LF: writing—original draft and investigation. GM: methodology, software, and formal analysis. ZL: project administration, validation, and data curation. BR: resources and supervision. HC: funding acquisition. All the authors have read and approved the final manuscript.

## Funding

This research was partly supported by the research fund from the Medical Sci-Tech Innovation Platform of Zhongnan Hospital, Wuhan University (PTXM2022020) and the Cultivate Plan of the Beijing Municipal Administration of Hospital (PX2018047).

## Conflict of interest

The authors declare that the research was conducted in the absence of any commercial or financial relationships that could be construed as a potential conflict of interest.

## Publisher's note

All claims expressed in this article are solely those of the authors and do not necessarily represent those of their affiliated organizations, or those of the publisher, the editors and the reviewers. Any product that may be evaluated in this article, or claim that may be made by its manufacturer, is not guaranteed or endorsed by the publisher.
